# I have to keep going for my kids (*Tengo que seguir adelante por mis hijos*): Migration, wellbeing and the collage narratives of mothers and children in transit

**DOI:** 10.1177/17455057261456862

**Published:** 2026-06-03

**Authors:** Madelyn Primeaux, Sarah Jay, Gulnaz Anjum, Halina Grzymała-Moszczyńska, Maria Elena Ramos Tovar

**Affiliations:** 1 8808 Department of Psychology, University of Limerick, Limerick, Ireland; 2 Department of Psychology, 6305 University of Oslo, Oslo, Norway; 3 Institute of Psychology, Ignatianum University in Krakow, Krakow, Poland; 4 School of Social Work and Human Development, 27771 Autonomous University of Nuevo León, Nuevo León, Mexico

**Keywords:** migrant mothers, children in transit, arts-based approaches, collage method, narrative inquiry, decolonial methodology, feminist health frameworks

## Abstract

**Background:**

Migration journeys profoundly affect women’s and children’s health, often producing layers of stress, trauma, and survival. Yet the experiences of mothers and children in transit remain underexplored, particularly through methods that allow them to narrate their own stories.

**Objectives:**

This study examines how migrant mothers and children in transit through northern Mexico use collaborative collage and storytelling to reflect on their lives and imagined futures, with attention to their health and wellbeing.

**Design:**

This qualitative study employed an arts-based research design. Collaborative collage-making and narrative inquiry were used to explore how migrant families reflect on and represent their experiences.

**Methods:**

Nineteen participants (nine mothers, ten children) residing in four migrant shelters engaged in arts-based sessions, creating collages and discussing their meanings. Data consisted of visual materials and audio-recorded conversations. NVivo software was used for coding and theme development during our reflexive thematic analysis.

**Results:**

Four themes shape this study: (1) What Mothers Carry centers on the emotional, physical and invisible weight mothers hold as they protect, nurture and attempt to preserve parts of themselves; (2) Hardship Beyond Borders captures the exposure to violence, hunger, and uncertainty families endured across countries, borders, and bureaucracies; (3) northern Mexico as Liminal Space reflects its role as a place of both protection and discrimination; and (4) A Future Imagined and Fought For highlights how mothers and children envision stability, education, and the chance to serve others.

**Conclusion:**

Collaborative collage created space for mothers and children to voice layered experiences of trauma, survival, and aspiration. These findings underscore the value of arts-based, participatory methods for illuminating women and children’s health and wellbeing in migration contexts and highlight the need for trauma-informed, culturally responsive support for families in transit. Grounded in the Mexican transit context, these findings are primarily intended to inform organizations and institutions working directly with migrant families in transit in northern Mexico. At the same time, the study aims to increase awareness among policy, academic, and public audiences by centering the humanity and lived experiences of mothers and children in forced migration.

## 1. Introduction

In recent decades, migration across the Americas has become increasingly politicized. The divide at the United States–Mexico border is where legal frameworks, surveillance, and public discourse often frame mobility as a threat rather than a right.^1^ These dynamics reflect not only contemporary policy shifts, but also historical and structural factors that shape who can move and under what conditions.

While the influx of Mexican immigrants has declined, the U.S. has now seen a rise of migrants from Central America, the Caribbean, and South America.^2^ Yet reaching the United States is rarely straightforward. For many families, northern Mexico becomes a forced place of transit as they make their journey north. While not meant to be their destination, it is a necessary stop and a part of their journey that is often prolonged, uncertain, and risky.^3^ In this context, transit migration refers to a temporary stay in a location that is not perceived as the migrant’s final destination, regardless of legal status.^4^ Recent policy shifts have intensified the stranding and vulnerability of transit families. Under the Biden administration, the May 2023 Circumvention of Lawful Pathways rule had already restructured access to asylum at the southern border, effectively requiring most migrants to secure a CBP One appointment or be presumed ineligible for protection.^5^ The January 20, 2025 suspension of CBP One under the second Trump administration removed even this constrained pathway, deepening pre-existing conditions of waiting and exposure. Against this instability, families in transit shared their stories with us.

In the following, we present the stories of migrant mothers and children in transit in northern Mexico. Using a reflexive thematic analysis grounded in a decolonial approach, and informed by a narrative lens, pieces of the lives of nine families, nine mothers and ten children, will be shared.^6,7^ In this paper, decolonial refers to a deliberate move away from dominant Western research traditions that prioritize objectivity, and individualism. Rather than imposing pre-defined categories, this study used open-ended conversations and participatory visual methods to allow families to represent their experiences in their own terms, recognizing the emotional and collaborative nature of knowledge production with mothers and children in transit. The results trace the weight mothers carry, the hardships families endure, northern Mexico as a space of waiting, and the futures they imagine. This study does not aim to generalize experiences or speak for all migrants. Rather, it seeks to center the voices of the mothers and children we had the privilege to learn from, to share their stories, foster connection, and reflect on the broader forces shaping their journeys.

### 1.1. Policy context and structural exclusions

For many migrants, the United States is imagined as a place of safety and opportunity, but this vision is shaped by contradiction. The “American Dream” narrative often obscures the violence, exclusion, and inequality that underpin U.S. immigration policy and its history of imperialism in Latin America.^8–10^ Recent policy shifts include the first Trump administration meeting their signature campaign pledge to ‘build a wall’ the length of the US-Mexican border, then in 2018 implemented zero tolerance border security including family separation and mass detention of migrants and asylum seekers from central and south America. The Biden administration has also been criticized for harsh border security including US Border Patrol agents on horseback preventing Haitian migrants crossing the Rio Grande.^11^

Families in this study relied on the U.S. Customs and Border Protection (CBP) One mobile application.^12^ CBP One functioned as a pathway for migrants to enter the U.S., often at the United States–Mexico border. While designed to create order, its limited capacity, lottery-style allocation, and access barriers left many unable to secure appointments. Even those who obtained appointments were not guaranteed entry, only the possibility of being processed at the border.^12^

The structural vulnerability associated with CBP One did not begin in January 2025. In May 2023, the Biden administration introduced the Circumvention of Lawful Pathways rule, which created a presumption of asylum ineligibility for migrants who entered the United States without first securing a CBP One appointment or seeking protection in a transit country. In effect, this transformed CBP One from one option among several into a de facto gatekeeping mechanism for asylum at the southern border. Limitations of this regulatory architecture, including restricted appointment capacity, technological and language barriers, and digital inequities, generated prolonged waiting periods and exposure to risk in northern Mexico well before the application’s suspension. Recognising this continuity is important for understanding the experiences described in this study, which unfolded against an institutionalised landscape of waiting that successive administrations have shaped rather than originated.

On January 20, 2025, following Donald Trump’s second inauguration — a presidency marked by anti-immigrant and anti-Latino rhetoric — CBP One was suspended.^13^ Within hours, nearly 30,000 scheduled appointments were cancelled, and an estimated 270,000 people were left without a pathway to entry.^5,12^ For participants in this study, the ending of CBP One reinforced their sense of limbo, where futures were indefinitely deferred and daily life reduced to waiting.

This suspension is not an isolated policy shift but part of a longer history of U.S. influence in Latin America. Economic policies, foreign interventions, and broader imperial relations have helped create the very conditions that drive migration.^8–11^ Recent qualitative research has examined how displaced mothers navigate wellbeing under uncertainty. Anjum and colleagues (2025) found that Ukrainian mothers in Norway experienced persistent limbo tied to temporary protection status, hindering future planning and threatening wellbeing.^14^ Yet research centering mothers and children in transit remains limited, particularly in the Americas where migration involves prolonged exposure to violence and shifting policy regimes. Recognizing this structural context underscores the need for decolonial and feminist methodologies that foreground migrant voices, understood here as approaches that challenge unequal power relations and dominant knowledge hierarchies in research with marginalized populations.^15^

### 1.2. Theoretical framework: Decolonial, feminist, and limbo perspectives

Research with migrant families requires frameworks that resist extractive and colonial traditions that have historically silenced or objectified marginalized communities. As Smith^16^ and Anjum and Aziz^17^ argue, decolonial approaches call for methodologies that are relational, flexible, and value lived experience, creativity, and community knowledge. Indigenous research paradigms, rooted in relational ontology, emphasize that knowledge is sculpted across time and relationships.^15,18^ Storytelling is central to these traditions, offering a means to articulate realities overlooked in Western epistemologies. In this study, “decolonial” refers not only to historical colonial domination, but to the ongoing hierarchies of power and knowledge that shape contemporary migration regimes, border governance, and whose experiences are recognized within research and policy. While stories are inevitably shaped by context and power, acknowledging these dynamics allows researchers to engage with them transparently and respectfully, rather than dismissing them as incomplete.

Building on this, narrative identity theory provides a psychological lens to explore how people construct meaning from past, present, and imagined futures.^7,19^ Yet its conventional assumptions of coherence, and continuity often do not reflect the fragmented, uncertain stories of migration. Families in transit shared narratives that were interconnected, shifting, and more focused on collective survival and hopes than on a singular, coherent self. Decolonial methodologies allow us to adapt narrative identity theory by valuing contradiction, relational meaning-making, and stories that remain unresolved.^19,20^

The concept of limbo adds a crucial dimension to this framework. As described by Anjum, Aziz, and Hamid,^21^ limbo is not a temporary pause but a psychosocial condition of suspended agency, prolonged uncertainty, and constrained wellbeing. For migrant mothers, limbo entails carrying the emotional and physical burdens of caregiving in contexts where futures are deferred and safety is precarious. A feminist lens further situates this condition as gendered: women’s health is shaped by the embodied labor of survival, where mothers balance protecting children, sustaining family hope, and negotiating their own needs.

Together, decolonial, feminist, and limbo perspectives underscore the importance of collage as more than a methodological choice: it became a practice of claiming space, resisting erasure, and making visible the layered health realities of families in transit. By combining narrative and visual expression, collage disrupted Western expectations of coherence and enabled mothers and children to articulate hardship, resistance and survival. This theoretical integration situates the findings within broader debates on women’s health, resistance, and justice, highlighting the need for trauma-informed and culturally grounded approaches to supporting families in conditions of limbo.

### 1.3. Methodological framework: Arts-based collage in migration research

Arts-based approaches offer powerful tools to explore the emotional and relational dimensions of migration, particularly when verbal expression alone cannot capture lived experience. Collage, the assembling of images, text, and materials into a visual narrative, provides participants with an accessible, creative medium to articulate memory, hardship, and hope.^22,23^ For children, collage reduces linguistic barriers and allows for symbolic expression, while for mothers, it creates space to share stories that may be difficult to disclose through traditional interviews. This participatory form of storytelling aligns with decolonial commitments by valuing layered, and affective ways of knowing.^18^

Previous research has shown that arts-based methods, including collage and participatory theatre, can illuminate complex migration experiences and intergenerational dynamics.^24–26^ Yet few studies have examined the shared meaning-making of mothers and children navigating transit together. This study addresses that gap by using collage not only as a method of data collection but also as a practice of connection, co-construction, resistance, and visibility. In this project, collage enabled migrant families in northern Mexico to reflect collectively on their lived experiences and imagined futures, generating both visual and narrative data that captured the realities of health, resistance, and survival in conditions of uncertainty.

### 1.4. Current study

Data collection for this study began three weeks after the suspension of the CBP One application, a decision that abruptly cancelled thousands of appointments and left many migrant families in a state of uncertainty. This policy shift, part of broader restrictive measures under the Trump administration, intensified the precarious conditions faced by mothers and children in transit. Against this backdrop, the project sought to document how families navigated the psychosocial burdens of displacement while also sustaining hope.

The original focus on families intending to reach the United States was broadened to include participants pursuing multiple migration trajectories. This adjustment reflected both the ethical responsibility to honor families’ willingness to share their stories and the methodological flexibility required when working with mobile, vulnerable populations.^27,28^ Sessions were conducted in northern Mexico, approximately three hours from the U.S. border, a key transit region where families often awaited or attempted to secure CBP One appointments. This context of political uncertainty and prolonged liminality shaped the families’ daily lives and framed the narratives that emerged during the study. This project was guided by the following research questions:

● How do migrant mothers and children in transit narrate their stories through collaborative collage and storytelling?
● What forms of health-related survival, burden, and intergenerational meaning-making emerge in these narratives?
● How does the experience of “limbo” influence the stories families tell about themselves?
● In what ways can arts-based, decolonial methods provide insights into women’s health and wellbeing that may be overlooked by conventional research approaches?

## 2. Methods

This study employed a qualitative, arts-based design to explore how migrant mothers and children in transit through northern Mexico reflected on their lives and imagined futures. Collaborative collage sessions, supported by guided conversation, generated both visual and narrative data. Consistent with decolonial and feminist commitments, the approach emphasized participant agency, relational meaning-making, and the co-construction of knowledge in contexts of uncertainty. This manuscript was prepared in accordance with the Consolidated Criteria for Reporting Qualitative Research (COREQ)^29^ checklist, provided as a separate supplementary file, to enhance transparency and rigor in reporting qualitative methods and findings (see supplemental file “COREQ checklist” for more details).

### 2.1. Research design

This study used a qualitative design informed by narrative theory and decolonial frameworks. We used arts-based research design to explore how migrant mothers and children in transit express their life experiences and feelings through collage in northern Mexico. Data included the collages participants created and the audio-recorded conversations that took place during each session. Photos were taken of each collage for analysis A total of 19 participants from nine families took part in the study. Families were from several countries in Latin America, in transit in northern Mexico, and residing in a migrant shelter at the time of the research. All sessions were led by the primary researcher, MP, and conducted in Spanish.

### 2.2. Ethical consideration

The study received ethical approval from the Faculty of Education and Health Sciences Research Ethics Committee at the University of Limerick under Project ID 2024_11_22_EHS. Data collection took place between February and April 2025. All participants and guardians provided verbal informed consent and assent prior to each session, which was documented through audio recording at the start of each session. Verbal consent was used in recognition of the heightened documentation risks that written consent can pose for migrant families in transit, a procedure approved by the ethics committee. This procedure was approved by the ethics committee in recognition of the heightened documentation risks that written consent can pose for migrant families in transit. Consent for publication of quotations and images of artwork was also obtained verbally from all participating mothers on behalf of themselves and their children. Pseudonyms were used to protect participants’ identities and identifying details were omitted. Sessions were intentionally designed to center participants’ feelings, emotions, and autonomy. A resource list was also provided with information on nearby shelters, medical services, and other available support.

Additional documentation supporting the study is provided in the supplementary file. This includes descriptive information for each participating family (Appendix A), the discussion guide used during the collage sessions (Appendix B), the full set of themes and codes developed during analysis (Appendix C), a sample of the artwork materials offered to participants (Appendix D), and the curated images participants could draw from when constructing their collages (Appendix E).

### 2.3. Participants

**Figure 1.**
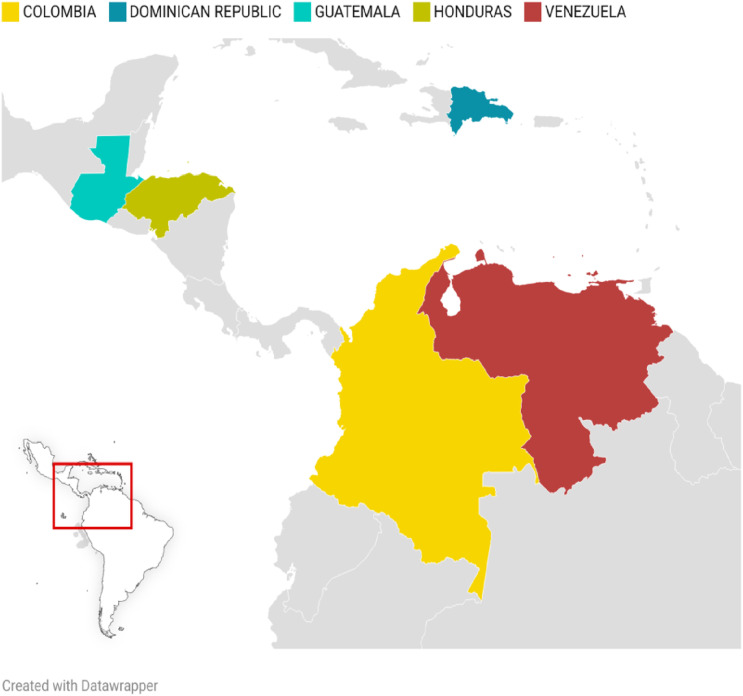
Countries of origin of participating families. *Note*. Alt Text: This map highlights in bold color the countries where participants originally came from: Dominican republic, Honduras, Guatemala, Colombia, and Venezuela.

The present study included mothers ages 29 to 43 and their children aged 8 to 12. There were nine families who took part, including three boys and seven girls. In total, there were 19 participants in the study, nine mothers and ten children. All participants that agreed to participate completed all sessions. In some sessions, additional children of participating mothers were present due to the shelter setting but did not take part in data collection. Families came from Honduras, Colombia, the Dominican Republic, Guatemala, and Venezuela, and were residing in four different migrant shelters in northern Mexico. See Figure 1 for a map of countries of origin. Intended destinations at the time of the study varied across families and included the United States, Canada, return to their country of origin, or remaining in northern Mexico. At the time of the sessions, families had been living in the shelters for two weeks to eight months. See Table 1 for Family Demographics and Supplementary Materials for descriptive information for each family.

**Table 1.** Participant demographics.

Variable	Subcategory	Frequencies and ranges
**Families**	Total families	9
	Mothers	9
	Children	10
Child Gender	Girls	7
	Boys	3
Child Age (years)	Range	8–12
Mother Age (years)	Range	29–43
**Country of Origin**	Honduras	3
	Venezuela	2
	Colombia	2
	Dominican Republic	1
	Guatemala	1
**Intended Destination**	United States	6
	Canada	1
	Mexico	1
	Return to country of origin	1
**Time Since Departure**	Less than 6 months	2
	6–12 months	3
	More than 1 year	3
	Unknown	1
**Time in Shelter**	Less than 90 days	5
	90 days or more	4

*Note.* Frequencies represent the number of families or individuals in each category. Ages are reported as ranges across participants.

### 2.4. Procedure

Participants were recruited from four migrant shelters in northern Mexico through posters and introductions by shelter staff, using a convenience sampling approach based on availability and willingness to participate. At the time of the study, MP was completing an internship at these shelters and had established relationships with the families, made possible through the prior partnerships developed by RT. She was trained in qualitative research methods, reflexive thematic analysis, cross-cultural communication, trauma informed care, and had previous field experience working with mothers and children in transit. Families were informed that participation was entirely voluntary and would not affect their access to services or their physical or psychological safety in any way. All participants were approached face to face. Eligibility was limited to mothers with children between the ages of 8-13, an age range shown to support reflective and creative participation in arts-based and narrative research.^30^ No families who expressed interest declined participation, and no participants withdrew from the study after providing consent. Sessions approximately lasted an hour, and a majority of participants agreed to three sessions. All sessions were audio recorded and collages were photographed for analysis. Brief observational and contextual notes were made during and after sessions to support interpretation of the data.

### 2.5. Collage sessions

Sessions took place in mother–child dyads, with one group involving a family of three (a mother and two children). Each session was guided by one or more time-based themes (past, present, and future), which served as flexible entry points for reflection rather than rigid categories of analysis. Rather than using a structured interview guide, sessions relied on open-ended prompts that supported participant-led storytelling and reflection. These prompts were not pilot tested due to the contextual constraints of conducting research with families in transit. Reflections could take the form of memories, feelings, or whatever participants felt was most meaningful to share. Although sessions were loosely organized around these prompts, the analysis focused on broader narratives of migration, wellbeing, and meaning-making.

Sessions were conducted in various locations, as it depended on the shelter’s availability. They took place in a chapel, outdoors, bedrooms and offices. Each session lasted approximately an hour, with the longest being two hours. At times, other family members, including siblings, were present during sessions due to caregiving responsibilities within the shelter setting; however, only the participating mother and child actively engaged in the collage sessions. Conversations accompanied each session and were audio recorded and used to contextualize the participants’ artwork. Brief, informal field notes were recorded following sessions to capture contextual details, researcher reflections, and observations that were not fully captured in the audio recordings. Families participated in multiple sessions across time when possible, corresponding to reflections on past, present, and future experiences. As families had already met the first researcher during her internship, there was a sense of familiarity and trust that often helped families open up and engage freely during the sessions.

Families first created individual collages, with a visual divider between the mother’s and child’s sections. After completing their individual sections, families were invited to collaborate on new, joint collage. This structure allowed for both personal reflection and joint expression, giving space to both distinct and collective narratives. In one case, the process was adapted to meet the needs of a child with autism. At the mother’s request, the session involved only individual collage-making, ensuring the format remained inclusive and comfortable for all participants. Each family completed all of their planned collages. The first author was constantly under the supervision of senior psychologists.

### 2.6. Artwork materials and visual data

**Figure 2.**
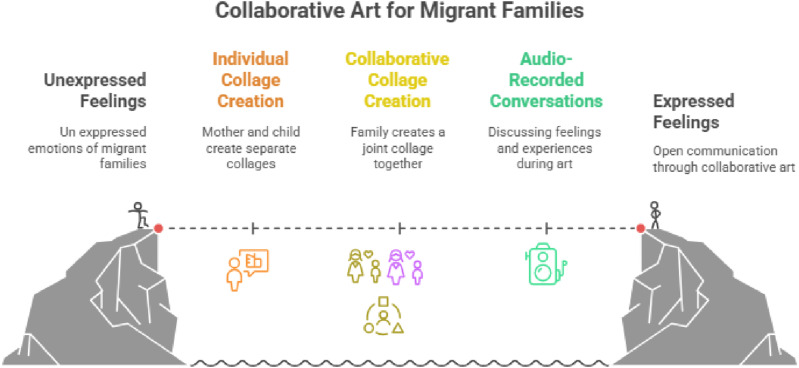
Art-based methods for understanding emotional experiences of migrant families. *Note*. This figure depicts the study’s arts-based methodology, showing how families moved from unexpressed to expressed feelings through individual collage creation, collaborative collage-making, and audio-recorded conversations. The bridge metaphor represents collaborative art as a pathway connecting internal experience to shared expression.

All materials for the artwork were provided and participants had complete freedom in their artistic choices. Collage was chosen specifically for its ability to convey nonverbal expression and invite participants to reflect and share their experiences in a personal and creative way. Especially as children may not be as comfortable verbally expressing their choices. Combining the visual and verbal data allowed for a layered understanding of mothers’ and children’s perspectives and emotions. Figure 2 provides a visual representation of the methods, while Table 2 presents materials families utilized to tell their stories.

**Table 2.** Materials provided for collage sessions.

Category	Description	Purpose
Basic art supplies	Scissors, glue sticks, markers, paint, pencils	Provided accessible tools for creating layered collages
Paper and surfaces	Colored construction paper, plain white paper	Offered flexibility and variation for collage backgrounds
Pre-printed images	Simple outlines of buses, cars, fences, and houses	Curated from prior research to ensure consistency across sessions while allowing individual interpretation^31–40^
Additional materials	Stickers, cutouts, small pieces of material	Added texture and creative variety to collages
Digital resources	Printer for participant-selected photos or images	Allowed families to include meaningful pictures, often of themselves or loved ones, supporting participant choice and representation

*Note.* All materials were provided by the research team and offered to participants at each session.

### 2.7. Reflexivity

All authors who contributed to this paper identify as females. The first author (MP)’s positionality as a Mexican-American Indigenous woman shaped every stage of this project. At the time of the study, MP had completed Bachelor’s Degree in Psychology and was pursuing her Master’s in the Psychology of Global Mobility, Inclusion, and Diversity in Society. Her academic background included training in qualitative research methods, reflexive thematic analysis, cross-cultural communication, and trauma-informed research practices, which informed both the design of the study and her engagement with participants. MP did not live with the fear of deportation, could move freely, and came from the very place many families aspired to reach. Balancing these power dynamics was not simple. Participants frequently asserted their own agency by asking questions, directing conversations, and sharing openly. Families were curious about life in the United States, MP’s background, and her work in northern Mexico. At times, participants apologized for speaking negatively about Mexico, assuming she might take offense, illustrating how her identity subtly shaped these interactions. Responding with care, openness, and validation helped foster trust and allowed conversations to unfold with greater ease.

All authors (a fulltime student, senior researchers, lecturers and professors) acknowledge that their identity, training, and privilege inevitably shaped this research, while remaining attentive to centering participants’ voices, meanings, and agency throughout the research process. All co-authors engaged with the data and findings through a lens of cultural sensitivity, recognizing that migration across the Americas is shaped by a colonial legacy in which the United States has played a central role in creating the conditions many families flee. This shared recognition informed the team’s commitment to a decolonial approach that honors families’ stories and the complexity of their hopes.

### 2.8. Data analysis

The analysis that follows is guided by Braun and Clarke’s six-phase approach to reflexive thematic analysis.^6,41^ To support the interpretation of participants’ reflections across time, McAdams’ theory of narrative identity^7^ was used, which understands life stories as a way people make meaning of their experiences. Although this study does not center identity, this lens helped frame how families used conversation and collage to tell stories about their lives and imagined future. Indigenous and decolonial approaches to storytelling that stress that narratives are nuanced, layered, and even fragmented aided the analysis.^18,20^ Like the collages themselves, these stories are a kind of patchwork. Inductive coding developed 77 codes, while 48 were used to build four overarching themes across the dataset. All coding and theme development were conducted by the first author, following an inductive and reflexive process grounded in the data. All coding was completed using NVivo 15. All sessions were conducted in Spanish, with ChatGPT-assisted translations carefully reviewed to preserve emotional intent and meaning. Both collage and conversation were treated as equally central to analysis. Transcripts were not returned to participants for review or correction. All coding and analysis was completed by the first author.

The collages conveyed symbolism and emotional depth often beyond words, while participants’ verbal reflections illuminated their creative choices. Nine families in transit through Mexico produced individual and shared collages, creating layered visual and verbal narratives shaped by movement, memory, and uncertainty. Through this integrated analysis of art and dialogue, four main themes and three subthemes emerged, each revealing distinct dimensions of the families’ experiences. While thematic saturation was reached during analysis, all participants who had agreed to take part were included to honor their willingness to share their stories and the ethical commitments of transit-based research.

Given the constraints of conducting research with families in transit, including unpredictable mobility and limited shelter stays, data collection continued with all consenting participants rather than being guided by a saturation-based stopping rule. During analysis, thematic saturation was considered achieved when no new themes emerged from continued engagement with the data. All participants who had consented were included in the final analysis to honor their willingness to share their stories, consistent with the ethical commitments of research with vulnerable, mobile populations. Data saturation was reached when no new themes emerged during analysis. Despite this, all planned sessions were completed to ensure inclusion of all participants who had consented to take part in the study.

## 3. Results

The analysis identified four themes that capture the layered experiences of migrant mothers and children in transit: (1) What Mothers Carry, (2) Hardship Beyond Borders, (3) Mexico as Liminal Space, and (4) A Future Imagined and Fought For. Across these themes, families revealed how exhaustion, protection, love, and hope interweave to sustain well-being in conditions of profound uncertainty.

Table 3 presents an overview of the four themes and their descriptions. Each theme is discussed below, illustrated by selected quotations and collage excerpts.

**Table 3.** Themes and descriptions.

Theme	Description	Subthemes (if applicable)
Theme 1. What Mothers Carry	Describes the emotional, physical, and relational burdens mothers carried while navigating migration, including loss, responsibility, caregiving, and efforts to preserve aspects of self.	1.1 Pain in partnership: widowhood, abandonment, betrayal, and strained partnerships. 1.2 Instincts to protect: emotional and physical strategies used to safeguard children. 1.3 Reclaiming selfhood: expressions of identity, desire, and individuality beyond motherhood.
Theme 2. Hardship Beyond Borders	Captures exposure to violence, hunger, dangerous journeys, loss, and institutional harm experienced across countries and borders.	Not subdivided; hardship was expressed across diverse and overlapping experiences.
Theme 3. Mexico as a Liminal Space	Reflects Mexico as a place of prolonged waiting characterized by uncertainty, ambivalence, and coexistence of support and exclusion.	Not subdivided; narratives reflected mixed and shifting experiences.
Theme 4. A Future Imagined and Fought For	Illustrates how families envisioned futures centered on stability, education, safety, and dignity despite prolonged uncertainty.	Not subdivided; future-oriented narratives emerged across families.

*Note.* Themes were developed through reflexive thematic analysis of collage and narrative data from nine families.

### 3.1. Theme 1. What Mothers Carry

Mothers held more than backpacks. They carried fear, hope, and exhaustion. This section holds some of the contradictions of motherhood: guilt and pride, grief and strength, pain and love. Three subthemes explore those layers: Pain in Partnership, Instincts to Protect, and Reclaiming Selfhood.

#### 3.1.1. Subtheme 1.1. Pain in partnership

Not all mothers made the journey alone, but many felt alone. Some had lost their partners, some left behind, and some were still with partners that betrayed them. Graciela made the journey alone as a widow, carrying the grief and responsibility of raising her five children alone. She shared, “They [her children] were really little when their dad died.” Flor made the journey alone as well, but under different circumstances: her husband abandoning her family for another woman. “Sometimes a person can do it alone, but in my case… I wish sometimes there was someone by my side. Some support.”

These excerpts show the weight of partnerships that are lost, broken, or strained, and the longing some may have for a steady hand beside them. While emotional pain in partnership left many mothers feeling vulnerable, motherhood also sparked fierce protectiveness— against both emotional wounds and physical threats.

#### 3.1.2. Subtheme 1.2. Instincts to protect

Protection takes different forms during the migration journey. Sometimes, it’s about shielding children from harsh realities. Other times, it means physical threats. When Itzel, Irene’s daughter, was asked what her present and future meant to her, she said, “To arrive in the United States” But in an earlier session, Irene said, “And they [the children] still dream of going to the U.S. And it pains me to bring them down from that cloud.” This dynamic is a depiction of the emotional weight of trying to protect children, while also managing their expectations.

For Flor, protection was more urgent and physical. Her decision to leave Honduras was not only about opportunity but meant protecting her daughter from gendered violence. “They [gang members] wanted to take her as their woman…the gang members were constantly trying to seduce her. Always saying things to her, always, and they would say to me, ‘Mother-in-law, she’s going to be my woman.’”

Yet amidst these acts of both survival and sacrifice, many women still held onto pieces of who they were before the migration trajectory. These pieces serve as reminders that these women are not only mothers, but people filled with histories, desires, and dreams.

#### 3.1.3. Subtheme 1.3. Reclaiming selfhood

Even as mothers endured displacement and the constant demands of survival, glimpses of their individuality surfaced in their words and collages. Graciela mentioned “I miss going out dancing.” While Dolores said, “[I want] to enjoy a lot of handbags, because I love them.” These reflections are powerful reminders of who these women were before migrating, and who they still are.

**Figure 3.**
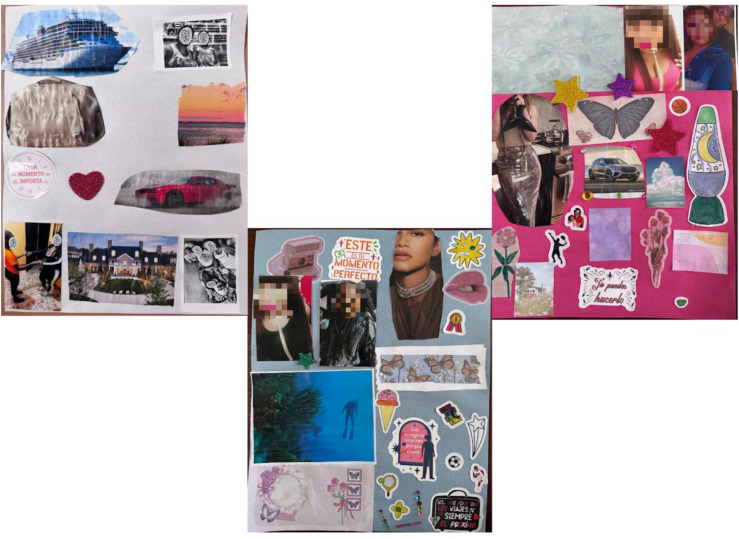
Collages of “What Mothers Carry”. *Note*. From left to right: Dolores’ future individual collage, elisa and Esmeralda’s joint future collage, Esmeralda’s individual future collage.

Esmeralda spoke most clearly about this. She references makeup in both an individual and joint collage. “This is because I like to dress that way. I don’t do it now, but I will later…I want to go back to doing that, because I love makeup and it’s part of my present, my past, and my future.” Her daughter Elisa also recognized this, including an image of lips in their joint collage, explaining “Because my mom likes it a lot.” These details suggest that children recognize their mothers’ individuality beyond caregiving roles. Figure 3 presents collages reflecting mothers’ efforts to preserve their identities amid displacement.

### 3.2. Theme 2. Hardship Beyond Borders

Pain followed families from home, across borders, and into an uncertain future. This theme explores the emotional burdens, hunger, political instability, and danger families experienced. When Dolores was asked why she left Colombia, she shared, “because of threats, attacks on our lives by armed groups. We can’t go back to our country. We just can’t.” Dolores’ words reveal how migration was not a choice but a response to fear and survival.

Not all hardships came in the form of violence or solely burdened mothers. Some took the shape of an aching absence. Gina, a 12-year-old, dedicated an entire collage to her dog left behind in Honduras. Her writing reads “I love you so much. I miss you. A kiss from a distance … I will always keep you in my heart wherever I am”. This intimate reflection demonstrates how this child’s connection to their pet is not trivial, but rooted in comfort, love, and longing for normalcy. In the face of instability, these attachments often represented how loss and memory shape children’s emotional landscapes.

For others, hardship intensified along the migration route. Irene reflected on her family’s journey through the Darien Gap: “We’ve suffered a lot on this journey. I want all of this to be worth it someday.” She described climbing steep slopes with her four children, soaked by days of rain. “We all slept in one tent that didn’t even fit us. One of my daughters said to me that ‘Mom, I should have stayed with grandma.’” She added quietly, “And we saw many people die.” Irene’s story suggests the physical and emotional tolls of migration, from witnessing danger through a screen to surviving it firsthand. Even after surviving the jungle, Irene reached northern Mexico only to find CBP One suspended, adding uncertainty to an already perilous journey.

“I thought, ‘Does it make sense to keep going?’ CBP One was over. We waited almost a year and never got through…We were so close, so close. And we didn’t make it. We uploaded our documents and then they closed the border.” Her words capture the doubt and uncertainty that define limbo, especially after CBP One was eliminated. Even after enduring one of the most dangerous stretches of the journey, families could still find themselves stalled by bureaucratic shifts that changed everything.

**Figure 4.**
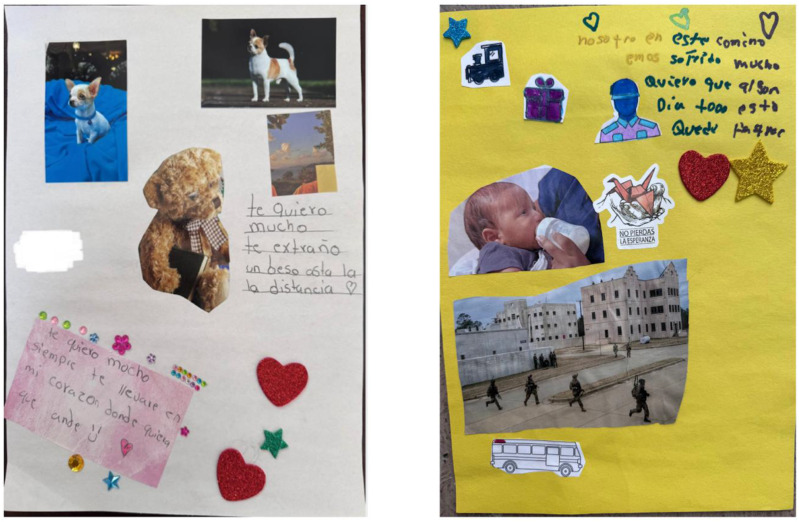
Collages of “Hardship Beyond Borders”. *Note*. From left to right: Gina’s Past Collage, Irene and Itzel’s Past Collage.

Other families encountered danger not just in the journey, but within the very system meant to provide aid. Beatriz’s family, for instance, experienced betrayal by those tasked with offering protection. Reflecting on her time in Mexico, she said, “And the same migration officials themselves sold us to the cartel.” Similarly, Esmeralda’s family suffered direct physical violence at the hands of Mexican immigration officers. Her 17-year-old son was beaten until he bled. She described her daughter fighting back: “Elisa confronted the immigration officer. And I had to fight with one of them too because she was telling the guards to shoot my son. As if we were at war.” Her account illustrates how quickly migration can shift from seeking safety to surviving violence. Figure 4 captures the emotional weight of loss and suffering experienced across the migration journey. Gina’s tribute to her dog left behind in Honduras and Irene and Itzel’s depiction of their arduous path reveal how hardship is held in memory and image.

### 3.3. Theme 3. Mexico as liminal space

For many families in this study, Mexico was meant to be a crossing point, not a long-term destination. Instead, it became a waiting room. In this theme, families express a mix of emotions about Mexico, from discomfort to gratitude. Families discussed the stress of adjusting, some were grateful for the safety Mexico provided, while others felt stuck. In one session, Gael reflected on his time at school and how he was bullied,“They say we’re from another country, that we say stuff… they say things. And that makes you feel bad. That’s why immigrants… we’re from another country; we’re migrating because of things they don’t know about. But still, they say stuff.”

His sister, Gina, wrote in her collage, as seen in Figure 6, “I don’t like Mexico because they are racist.” The siblings’ accounts reflect experiences of exclusion and discrimination that shaped their sense of belonging in Mexico. Still, not all reflections were entirely negative. Camila said: “You never expect to be in a situation like this… But, well, you can only do what’s possible…So, despite the situation we’re in and what we’re going through, we don’t lose hope that things will get better.”

**Figure 5.**
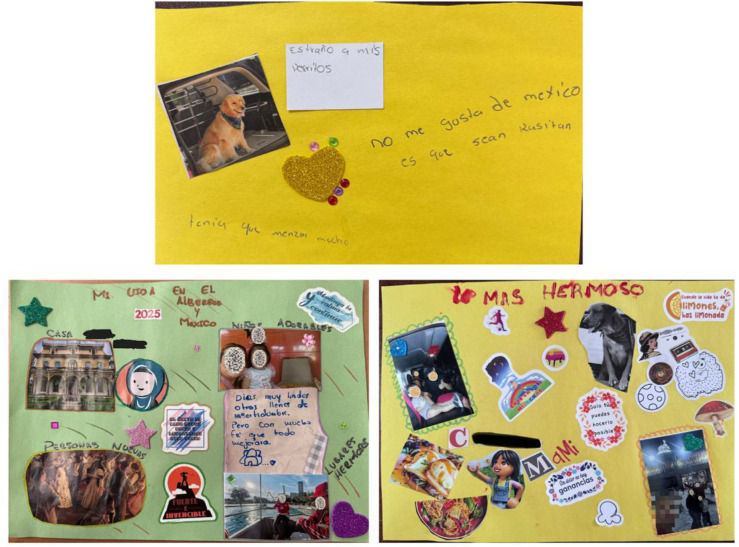
Collages of “Mexico as a liminal space”. *Note*. From top to bottom, left to right: Gina’s present collage, camila’s present collage, and camila and cesar’s joint present collage.

Her son, Cesar added, “Mommy, when life gives you lemons, make lemonade. And that’s what we’re going to do now.” Later on Camila stated, “The Mexican people have shown us not only generosity… …helping us, putting food on the table, making sure we’re not missing anything, making sure the kids are okay, opening the doors to education so they can study. I’m eternally grateful. You don’t really grasp it… until you live it.” These moments from Camila and Cesar show that gratitude and hope can coexist with frustration. Camila’s appreciation for Mexico and the shelter highlights the vital support these spaces provide (see Figure 5).

### 3.4. Theme 4. A Future imagined and fought for

**Figure 6.**
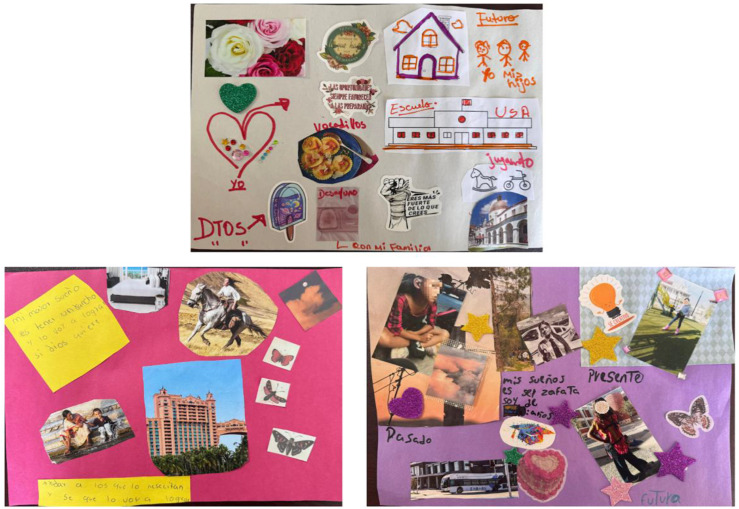
Collages of “A future imagined and fought for”. *Note*. From top to bottom, left to right: Flor’s future collage, Gina’s future collage, and Hayley’s combined collage.

Even when the future felt far away, families still imagined it. They dreamed of stability, school, careers, and peace. These dreams weren’t abstract but made tangible with intention. Hayley shared, “In the future, my dream is to be a flight attendant. I’d like to catch a flight to Venezuela. Have my 15^th^ birthday party, graduate, and volunteer too when I’m a teenager.” Her words reflect a desire to return home and maintain cultural connections. Gina had similar ideas of volunteering, writing in Figure 6, “To help those who need it and I know I will do it.” Such statements suggest a shift from survival toward imagining future contributions.

Mothers also reflected on the future through care and perseverance. Irene shared, “And all my dreams… you know? They’re connected to that. To have my home again and for all of us to be stable, so we’re not suffering so much. Not so much hunger.” Amanda admitted, “Yes, I’m afraid we won’t achieve what we want to achieve. That’s the fear.” Yet, hope persisted. As Flor reflected on her collages, “…I’ve written it down and someday it will come true.” Across families, the future was imagined not as an escape but as restoration, a return to safety, dignity, and belonging.

Figure 6 presents collages depicting families’ aspirations for stability, education, and contribution. Flor’s vision of home and school, Gina’s commitment to helping others, and Hayley’s dream of becoming a flight attendant reflect forward-looking hope amid uncertainty.

## 4. Discussion

This project explored how migrant mothers and children in transit shared their narratives through collage and conversation. Their stories revealed how wellbeing is shaped within uncertainty, care, and daily acts of endurance. In response to the first research question, the collages showed that both mothers and children used creativity to express emotion, memory, and determination. Mothers showed how their emotional and physical health were tied to their children while children’s participation gave them space to share what it means to grow up in movement. Together, their work reflected what it takes to hold a family together in uncertainty.

Findings of theme 1 align with emerging research on the burdens of displaced motherhood. Anjum and colleagues found Ukrainian refugee mothers in Norway experienced persistent tension between prioritizing children’s stability and managing their own constrained futures under temporary protection.^14^ These findings are also aligned with Ukrainian refugee mothers in Poland.^42^ Similarly, Dillon and Ali’s collage-based study with transnational mothers documented the “in-between space” where women navigate competing demands of caregiving and self-preservation.^24^ However, the present study extends this literature by documenting how mothers in *transit*, actively work to preserve aspects of selfhood while navigating survival. This dimension of identity maintenance amid displacement has received limited attention in forced migration research, which tends to focus on trauma and loss rather than ongoing self-expression.

The second research question focused on survival, burden, and meaning across generations. Mothers spoke about exhaustion. Fear and the challenge of protecting their children while facing so much hardship themselves. Irene shared the pain of lowering her children’s expectations about reaching the United States and Flor described leaving Honduras as an act of defense for her daughter. Their words show how motherhood in migration is shaped by constant vigilance and emotional strain. Children carried their own forms of survival, often expressed through love and memory.

Cumulative hardships documented in theme 2 converge with research on compounding risks faced by migrants traversing Latin America. Aguilar and colleagues documented the dangers concentrated along Central American and Mexican transit routes, including violence, exploitation, and institutional harm.^3^ Maioli and colleagues, studying unaccompanied migrant youth in Mexican shelters, similarly found that violence and poverty led to loss of crucial formative years, with resilience maintained through cultural narratives of hope and family responsibility.^26^ Our findings extend this work by illustrating how hardship is experienced *relationally* within families: children’s losses (a pet left behind, a grandmother missed) are held alongside mothers’ fears, and family members, including young children, become active participants in protection. This intergenerational dimension of cumulative trauma warrants further investigation in migration health research.

The third research question considered how life in limbo shaped families’ wellbeing. For most, Mexico was both a place of safety and a source of stress. Gina and Gael spoke of discrimination and bullying, showing how exclusion can affect a child’s sense of belonging. At the same time, other families found comfort and care in small moments of support. When Camila told her son about the difficulty of their situation, he tried to make light of it. Their exchange captured what many families expressed, a way of holding frustration and hope at once. Waiting in Mexico was not just a pause but an emotional state that affected health, creating tiredness, uncertainty, and tension. Yet even while experiencing life in limbo, families found ways to connect and create together. These moments became small acts of care, helping to maintain stability when everything else felt temporary. Since the completion of this study, many of the participating families have returned to their countries of origin due to the suspension of migration pathways and worsening political conditions. Their return underscores how quickly migration routes shift and how deeply a family’s wellbeing depends on policies that are consistent, humane, and family-centered.

Findings of theme 3 show ambivalence families expressed toward Mexico, it echoes conceptualizations of limbo as a psychosocial condition of suspended agency. Anjum, Aziz, and Hamid described limbo as an institutionally produced state where uncertainty about the future exacerbates psychological stress and constrains life-planning.^21^ The suspension of CBP One in January 2025 intensified a condition that the Circumvention of Lawful Pathways framework had already produced, leaving families, as Médecins Sans Frontières documented, stranded without pathways forward.^5^ Our findings confirm this framing while adding a developmental dimension: children like Gael and Gina encountered limbo not only temporally but socially, through peer discrimination and experiences of being “othered” in schools. Notably, participants’ experiences of gratitude coexisting with frustration suggest that limbo is not uniformly negative but characterized by contradictory emotions.

The final research question asked how arts-based and decolonial methods can highlight aspects of health and wellbeing often overlooked. Collage gave participants control over what to share and how to share it. For mothers, it became a space to remember themselves as people beyond caregiving. Graciela talked about missing dancing. Dolores described her love for handbags. Esmeralda included makeup in her collage, and her daughter Elisa added an image of lips, recognizing and celebrating her mother’s identity. These details reveal how expression and self-recognition are tied to wellbeing. Including children in this process was also important. While many researchers avoid working with refugee children due to ethical concerns, this project shows that creative, flexible methods allow children to share their perspectives safely and meaningfully. Their collages and words deepened understanding of family life and demonstrated that children can reflect on migration in ways that are honest and emotionally safe.

The future-oriented narratives in this study challenge deficit-focused frameworks which position displaced families primarily as traumatized victims. Children’s aspirations to become flight attendants, to volunteer, and to “help those who need it” reflect what Maioli and colleagues termed “resistance” among migrant youth in Mexico, a forward-looking agency maintained through faith, hope, and family responsibility.^26^ Similarly, Anjum and colleagues found that Ukrainian refugee mothers maintained motivation and adaptive resilience despite institutional barriers, actively working toward integration and stability.^14^ Our findings extend this by demonstrating that future-making is an intergenerational practice: mothers and children imagine futures *together*, with children’s dreams often centered on returning home or contributing to others. This prosocial orientation has implications for how humanitarian programs engage young migrants, not merely as recipients of aid but as future contributors.

A key strength of this study was the trust established with participating families. The first author’s prior relationship with families through her internship at the shelters created a sense of familiarity that made participation feel natural and collaborative. The combination of conversation and collage revealed how emotional and physical dimensions of health and wellbeing are deeply interconnected for mothers and children in transit. Importantly, this study demonstrates that including children in migration research is both possible and necessary when approached with care, flexibility, and age-appropriate methods. Arts-based approaches allowed children to express experiences and perspectives through visual means, even when they were less verbally active than their mothers.

### 4.1. Limitations

Several limitations should be considered when interpreting the findings. First, the application of decolonial methodologies was constrained by the realities of transit research. Although participants interpreted their artwork during sessions, they were not involved in coding, theme development, or reviewing transcripts and findings. Member checking and co-analysis were infeasible given participants’ geographic mobility, uncertain trajectories, and time-limited shelter stays. Some families had already left Mexico or returned to their countries of origin before analysis was complete. Future research might explore remote or asynchronous methods for sustaining participant engagement across dispersed locations.

Second, caregiving responsibilities shaped participation during collage sessions. Many mothers were caring for multiple children simultaneously. Many were breastfeeding, providing snacks, or tending to younger siblings, which at times limited their ability to fully engage. In some cases, the participating child also assumed caregiving responsibilities for siblings. Providing childcare support may have allowed for fuller engagement in future studies.

Third, our focus on mother-child dyads does not capture the full range of caregiving arrangements during forced displacement. Children often flee with grandmothers, aunts, or neighbors who assume primary caregiving roles, sometimes caring for multiple children simultaneously. These configurations carry distinct implications for children’s sense of security, as separation from parents represents an additional rupture from their previous lives. Fathers and older siblings who serve as primary caretakers are also absent from this analysis.

Finally, the shared Spanish language between participants and the Mexican host community may have eased daily navigation and research participation, but it also limits the transferability of these findings. Families displaced into linguistically unfamiliar contexts. For instance, in the contexts where they cannot communicate with service providers, authorities, or host communities they may face additional layers of isolation and disorientation that were not captured here.

### 4.2. Applications and further research

Current health and migration policies often treat women and children separately, dividing care into maternal and child health rather than recognizing the family as a shared space of wellbeing. The findings from this study challenge the divide. Health must be approached relationally, through policies that see connection, protection and mutual care as central to wellbeing in movement. Family centered models can inform how shelters, clinics, and humanitarian programs support emotional and social health together.

Further research could explore how families’ stories shift in the next step of their journey and how changing migration policies continue to shape their sense of health and belonging. It would also be valuable to study how art-making fosters relational wellbeing between mothers and children. During our final session, Flor reflected that creating alongside her children “gave me freedom… it’s precious.” Her words capture how collaborative spaces can support both expression and connection, reminding us that health in migration is not only about survival but also about moments of calm, togetherness, and care.

## 5. Conclusion

We demonstrate how collaborative collage and storytelling provided migrant mothers and children in transit with a space to articulate experiences that were fragmented, and emotional. These narratives illuminated not only the hardships of displacement but also the strengths families drew from memory, intergenerational ties, and imagining the future. They revealed the maternal burden of caregiving under conditions of precarity, while also highlighting children’s agency and shared hopes for stability, belonging, and wellbeing. By foregrounding participants’ narratives, the project underscores the value of arts-based, decolonial methods for women’s health research. Collage served as both an expressive tool and a therapeutic practice, offering insight into the intersections of migration, maternal identity, and child wellbeing. These stories signal the need for trauma-informed, culturally responsive, and participatory approaches in health practice and policy. As we reflect on these narratives, they remind us that this research is not only about documenting experience but about sharing lived realities. Families’ experiences should guide interventions and remind us that health equity depends on amplifying the voices of those most affected.

## Supplemental material

Supplemental material - I have to keep going for my kids (*Tengo que seguir adelante por mis hijos*): Migration, wellbeing and the collage narratives of mothers and children in transit Supplemental material for I have to keep going for my kids (*Tengo que seguir adelante por mis hijos*): Migration, wellbeing and the collage narratives of mothers and children in transit by Madelyn Primeaux, Sarah Jay, Gulnaz Anjum, Halina Grzymała-Moszczyńska and Maria Elena Ramos Tovar in Women’s Health.

Supplemental material - I have to keep going for my kids (*Tengo que seguir adelante por mis hijos*): Migration, wellbeing and the collage narratives of mothers and children in transit Supplemental material for I have to keep going for my kids (*Tengo que seguir adelante por mis hijos*): Migration, wellbeing and the collage narratives of mothers and children in transit by Madelyn Primeaux, Sarah Jay, Gulnaz Anjum, Halina Grzymała-Moszczyńska and Maria Elena Ramos Tovar in Women’s Health.

## Data Availability

We have provided supplemental information and additional details for this paper along with the submission. Additional anonymized data will be provided upon request. Please reach out to the corresponding author.*

## References

[1] Bersin A Bruggeman N Rohrbaugh B . Migration at the U.S.–Mexico border: a challenge decades in the making. Migration Policy Institute, 2024. Available from. https://www.migrationpolicy.org/sites/default/files/publications/mpi-border-history-report-2024_final.pdf

[2] Moslimani M Passel JS . Key findings about U.S. immigrants. Pew Research Center, 2024. Available from. https://www.pewresearch.org/short-reads/2024/09/27/key-findings-about-us-immigrants/

[3] Aguilar M Álvarez L Aragón E . Riesgos y protección en las zonas más peligrosas de las rutas de tránsito migratorio por Centroamérica y México. San José (CR): Organización Internacional para las Migraciones (OIM), 2024.

[4] Testa G . Transit migration and development (KNOMAD Paper No. 52). Mixed Migration Centre, 2023. Available from. https://mixedmigration.org/wp-content/uploads/2024/01/309_KNOMAD_52_Transit_Migration_Development.pdf

[5] Médecins Sans Frontières . Lives in limbo after CBP One closure in Mexico. : Doctors Without Borders, 2025. Available from: https://www.doctorswithoutborders.org/latest/lives-limbo-after-cbp-one-closure-mexico

[6] Braun V Clarke V . Conceptual and design thinking for thematic analysis. Qual Psychol 2022; 9(1): 3–26. 10.1037/qup0000196

[7] McAdams DP . Narrative identity. In: Schwartz SJ Luyckx K Vignoles VL (eds). Handbook of identity theory and research. Springer Science + Business Media, 2011, pp. 99–115. 10.1007/978-1-4419-7988-9_5

[8] Coronado JD . Manmade immigration crisis caused by U.S. intervention in Latin America. NEXO. 2019 Spring. Available from. https://jsri.msu.edu/publications/nexo/vol-xxii/no-2-spring-2019/manmade-immigration-crisis-caused-by-u-s-intervention-in-latin-america.22 2.

[9] Páez T . Amid economic crisis and political turmoil. Venezuelans form a new exodus. Migration Policy Institute, 2017. Available from. https://www.migrationpolicy.org/article/amid-economic-crisis-and-political-turmoil-venezuelans-form-new-exodus

[10] Seelke CR Nelson RM Margesson R , et al. Venezuela: background and U.S. relations (CRS Report No. R44841). Congressional Research Service, 2022. Available from. https://crsreports.congress.gov/product/pdf/R/R44841

[11] Timothy BG . Local support for the US–Mexico border wall and local immigration policy. Territory, Politics, Governance 2024; 12(7): 948–968. 10.1080/21622671.2022.2095009

[12] American Immigration Council . CBP One: An overview [Fact sheet]. : American Immigration Council, 2025. Available from: https://www.americanimmigrationcouncil.org/research/cbp-one-overview

[13] Canizales SL Agius Vallejo J . Latinos & racism in the Trump era. Dædalus. 2021 Spring; American Academy of Arts & Sciences, Available from: https://www.amacad.org/publication/daedalus/latinos-racism-trump-era

[14] Anjum G Isaac L Grzymała-Moszczyńska H , et al. “My suitcases are still not fully unpacked”: Ukrainian refugee mothers under Norwegian temporary collective protection. Int J Qual Stud Health Well-being 2025; 20(1): 2580074. 10.1080/17482631.2025.2580074 41236358 PMC12621327

[15] Chilisa B . Indigenous research methodologies. Sage Publications, 2012.

[16] Smith LT . Decolonizing methodologies: research and indigenous peoples. 2nd ed. Zed Books, 2012.

[17] Anjum G Aziz M . Advancing equity in cross-cultural psychology: embracing diverse epistemologies and fostering collaborative practices. Front Psychol 2024; 15: 1368663. 10.3389/fpsyg.2024.1368663 38638521 PMC11024300

[18] Aziz M Anjum G Nawaz AR , et al. Who speaks for climate migrants? A justice-oriented bibliometric analysis of Global South research. Front Climate 2025; 7: 1658517. 10.3389/fclim.2025.1658517

[19] McAdams DP . The psychology of life stories. Rev Gen Psychol 2001; 5(2): 100–122. 10.1037/1089-2680.5.2.100

[20] Fadel L . Re-search on the hyphen: (Re)writing the fragmented self within contexts of displacement. Genealogy 2023; 7(4): 80. 10.3390/genealogy7040080

[21] Anjum G Aziz M Hamid HK . Life and mental health in limbo of the Ukraine war: how can helpers assist civilians, asylum seekers and refugees affected by the war? Front Psychol 2023; 14: 1129299. 10.3389/fpsyg.2023.1129299 36874809 PMC9983366

[22] Barron A . Collage as method. In: Rodekirchen M Pottinger L Briggs A , et al. (eds). Methods for Change Volume 2: Impactful social science methodologies for 21st century problems. Aspect and The University of Manchester, 2023.

[23] Nathan S Hodgins M Wirth J , et al. The use of arts-based methodologies and methods with young people with complex psychosocial needs: a systematic narrative review. Health Expect 2023; 26(1): 88–101. 10.1111/hex.13705 PMC10010092 36628644

[24] Dillon AM Ali T . Collage-based narratives of mothers of third culture kids: the in-between space of transnational families in the UAE. Front Sociol 2024; 9: 1497479. 10.3389/fsoc.2024.1497479 39624615 PMC11611520

[25] Kaptani E Erel U O’Neill M , et al. Methodological innovation in research: participatory theater with migrant families on conflicts and transformations over the politics of belonging. J Immigr Refug Stud 2021; 19(1): 68–81. 10.1080/15562948.2020.1843748

[26] Maioli SC Devakumar D Berenzon Gorn S , et al. Growing up in transit: personal development and resistance of migrant adolescents travelling through Mexico unaccompanied. J Migr Health 2024; 10: 100245. 10.1016/j.jmh.2024.100245 40103921 PMC11915520

[27] Liamputtong P . Flexible and collaborative investigative methods. Flexible and collaborative investigative methods. : Sage Publications, 2007, pp. 118–138. 10.4135/9781849209861.n6

[28] Anjum G Aziz M . Climate change and gendered vulnerability: a systematic review of women’s health. Womens Health (Lond) 2025; 21: 17455057251323645. 10.1177/17455057251323645 40071991 PMC11905046

[29] Tong A Sainsbury P Craig J . Consolidated criteria for reporting qualitative research (COREQ): a 32-item checklist for interviews and focus groups. Int J Qual Health Care 2007; 19(6): 349–357. 10.1093/intqhc/mzm042 17872937

[30] Scionti N Zampini L Marzocchi GM . The relationship between narrative skills and executive functions across childhood: a systematic review and meta-analysis. Children (Basel) 2023; 10(8): 1391. 10.3390/children10081391 37628390 PMC10453360

[31] Marchevska E Defrin C . Reframing migrant narratives through arts practice. Arts (Basel) 2023; 12(2): 58. 10.3390/arts12020058

[32] Brown A Spencer R McIsaac JL , et al. Drawing out their stories: a scoping review of participatory visual research methods with newcomer children. Int J Qual Methods 2020; 19: 1609406920933394. 10.1177/1609406920933394

[33] Garriba F . The healing journey of forcibly displaced children through art. UNHCR 2024, Available from: https://www.unhcr.org/ng/news/stories/healing-journey-forcibly-displaced-children-through-art

[34] UNICEF . Child alert: children on the move in Latin America and the Caribbean. : UNICEF, 2023. Available from: https://www.unicef.org/media/144741/file/Migration-Child-Alert-English-2023.pdf

[35] UNICEF . Children’s drawings illustrate the perils of the Darien jungle. UNICEF, 2022. Available from: https://www.unicef.org/lac/en/childrens-drawings-illustrate-the-perils-of-darien-jungle

[36] Kronick R Rousseau C Cleveland J . Refugee children’s sandplay narratives in immigration detention in Canada. Eur Child Adolesc Psychiatry 2018; 27(4): 423–437. 10.1007/s00787-017-1012-0 28643110

[37] Brown PL . Making art at the border: “My way of showing that I’m still here”. New York Times 2019, Available from: https://www.nytimes.com/2019/07/19/arts/art-detention-centers-migrants.html

[38] Clacherty G Art-based . narrative research with unaccompanied migrant children living in Johannesburg, South Africa. J Borderl Stud 2021; 36(4): 547–563. 10.1080/08865655.2019.1621766

[39] Farokhi M Hashemi M . The analysis of children’s drawings: social, emotional, physical, and psychological aspects. Procedia Soc Behav Sci 2011; 30: 2219–2224. 10.1016/j.sbspro.2011.10.433

[40] Thompson F . Children with toys carried to safety as migrant crossings resume. Press Association, 2022. Available from: https://glucksman.idm.oclc.org/login?url=https://www.proquest.com/wire-feeds/children-with-toys-carried-safety-as-migrant/docview/2673559938/se-2

[41] Braun V Clarke V . Using thematic analysis in psychology. Qual Res Psychol 2006; 3(2): 77–101. 10.1191/1478088706qp063oa

[42] Baran M Grzymała-Moszczyńska H Zjawińska M , et al. Superhero in a skirt: Psychological resilience of Ukrainian refugee women in Poland. A thematic analysis. Int J Clin Health Psychol 2024; 24(4): 100506. 10.1016/j.ijchp.2024.100506 39823096 PMC11735986

