# Supplementary material for: I have to keep going for my kids (Tengo que seguir adelante por mis hijos): Migration, wellbeing and the collage narratives of mothers and children in transit
Source: Womens Health (Lond). 2026 Jun 3;22:17455057261456862. doi: 10.1177/17455057261456862 (PMC13237257; doi:10.1177/17455057261456862)
Supplement: Supplemental material - I have to keep going for my kids (Tengo que seguir adelante por mis hijos): Migration, wellbeing and the collage narratives of mothers and children in transit [file sj-pdf-1-whe-10.1177_17455057261456862.pdf]

COREQ Checklist

1. MP conducted the discussions and collage-making sessions. She was constantly under the supervision of senior psychologists – the coauthors. (Section: Collage Sessions/ Page no. 17)
2. At the time of the study, MP had a completed Bachelor's Degree in Psychology and was pursuing her Master's in the Psychology of Global Mobility, Inclusion, and Diversity in Society. (Section: Reflexivity/ Page no. 19)
3. MP's occupation at time of the study was a full-time student. Other authors are Senior researchers, lecturers and professors. (Section: Reflexivity/ Page no. 19)
4. All authors who contributed to this paper identify as females. (Section: Reflexivity/ Page no. 19)
5. MP has been trained in qualitative research methods, reflexive thematic analysis, cross-cultural communication, trauma informed care, and had previous field experience working with mothers and children in transit. (Section Procedures/ Page no. 15)
6. MP had established prior relationships with participants, as she visited the shelters and volunteered before conducting research. (Section Procedures/ Page no. 15)
7. Participants were made aware that MP was conducting this research project as a part of her Master's program and aimed to understand the experiences of mothers and children in transit. They were aware of her background, including her Mexican American and Indigenous identity and family migration history. (Section: Reflexivity/ Page no. 19)
8. The researcher acknowledged her positionality and interest in migration and family wellbeing and maintained reflexivity through open-ended questions, participant-led conversations, self-reflection, and supervisory discussions to minimize assumptions and bias. (Section: Reflexivity/ Page no. 19)
9. The study used a qualitative design informed by narrative theory and decolonial frameworks. (Section: Research Design/ Page 10)
10. Participants were recruited through convenience sampling at migrant shelters, with the assistance of shelter staff who helped introduce the study to eligible families. Participation was voluntary. (Section: Procedures/ Page no. 15)
11. Participants were approaching face to face. (Section: Procedures/ Page no. 15)
12. There were 19 participants in the study, nine mothers and ten children. (Section: Participants/ Page no. 12)
13. All participants that agreed to participate completed all agreed to sessions. (Section Participants/ Page no. 12)
14. Data was collected at the shelters in any free and private space. (Section Procedures/ Page no. 15)
15. In some sessions, additional children of participating mothers were present due to the shelter setting but did not take part in data collection. (Section Participants/ Page no. 12)
16. Inclusion criteria included migrant mothers and their accompanying children ages 8-12 and currently in transit in Mexico. (Section Participants/ Page no. 12)

17. Yes. Sessions were prompted by time-based themes (past, present, future) and supported by guided conversation during collage making. The prompts were used as flexible entry points rather than strict categories of analysis. (Section Procedures/ Page no. 15)
18. No repeat interviews were conducted. Data collection consisted of multiple planned sessions with participating mothers and children as part of the study design. (Section: Research Design/ Page 10)
19. Sessions were audio recorded and collages were photographed for analysis. (Section Procedures/ Page no. 15)
20. Brief observational and contextual notes were made during and after sessions to support interpretation of the data. (Section: Procedures/ Page no. 15)
21. Sessions approximately lasted an hour, and a majority of participants agreed to three sessions. (Section: Procedures/ Page no. 15)
22. Data saturation was reached when no new themes emerged during analysis. Despite this, all planned sessions were completed to ensure inclusion of all participants who had consented to take part in the study. (Section: Data Analysis/ Page no. 21)
23. Transcripts were not returned to participants for review or correction. (Section: Data Analysis/ Page no. 20)
24. One researcher, MP, conducted all of the data coding. (Section: Data Analysis/ Page no. 20)
25. The analytic process from initial codes to final themes was documented narratively. Codes were developed inductively through repeated engagement with the data and iteratively grouped into broader themes. A formal coding tree diagram was not created. (Section: Data Analysis/ Page no. 20)
26. All themes were derived from the data. (Section Data Analysis/ Page no. 20)
27. NVivo software was used to analyze and manage data. (Section: Data Analysis/ Page no. 20)
28. Participants did not provide feedback. (Section: Data Analysis/ Page no. 20)
29. Participant quotations and artwork were used to illustrate the findings. Quotations and collages were identified using pseudonyms and participant type (mother or child). (Section: Data Analysis/ Page no. 20)
30. Data presented and findings were consistent. (Section: Discussion/ Page no. 33)
31. Major themes were clearly present. (Section: Results/ Page no. 21)
32. There was a discussion on minor themes. (Section: Discussion/ Page no. 36)

Tong A, Sainsbury P, Craig J. Consolidated criteria for reporting qualitative research (COREQ): a 32-item checklist for interviews and focus groups. International Journal for Quality in Health Care. 2007. Volume 19, Number 6: pp. 349 – 357.
