# Supplementary material for: I have to keep going for my kids (Tengo que seguir adelante por mis hijos): Migration, wellbeing and the collage narratives of mothers and children in transit
Source: Womens Health (Lond). 2026 Jun 3;22:17455057261456862. doi: 10.1177/17455057261456862 (PMC13237257; doi:10.1177/17455057261456862)
Supplement: Supplemental material - I have to keep going for my kids (Tengo que seguir adelante por mis hijos): Migration, wellbeing and the collage narratives of mothers and children in transit [file sj-pdf-2-whe-10.1177_17455057261456862.pdf]

Supplementary Materials

Table of Contents

Appendix A. Descriptive Information for Each Family

Appendix B. Discussion Guide

Appendix C. Themes and Codes

Appendix D. Sample of Materials

Appendix E. Curated Images

Appendix A

Descriptive Information for Each Family

Family A consisted of a mother (Amanda, 32) and daughter (Andrea, 11) from Honduras. They left their country of origin in August 2024 and have been residing in a shelter for eight months, along with Andrea's younger brother. Amanda is a single mother, and the family aims to resettle in the United States. Amanda and Andrea participated in three separate sessions (past, present, and future) and completed a total of nine collages across individual and joint formats. Their quotes and/or collages can be found in theme 4.

Family B consisted of a mother (Beatriz, 36) and daughter (Bianca, 8) from Honduras. They left home in May of 2024 and have been residing in a shelter for 18 days, along with Beatriz's husband, younger daughter, and older son. As the younger daughter is a toddler and joined in the sessions, Beatriz was often tending to her as well. Family B aims to stay in Mexico but further west. Beatriz and Bianca participated in three separate sessions (past, present, and future) and completed a total of nine collages across individual and joint formats. Family B's quotes and/or collages can be found in the title and theme 2.

Family C consisted of a mother (Camila, 32) and son (Cesar, 8) from Colombia. They left Colombia in October of 2024 and have been living in a shelter for one month, with Camila's husband and older son. Cesar and Camila aspire of resettling in Canada. They participated in three separate sessions (past, present, and future) and completed a total of nine collages across individual and joint formats. Their quotes and/or collages can be found in theme 3.

Family D consisted of a mother (Dolores, 29) and daughter (Dulce, 12) from Colombia. They left Colombia in January of 2025 and have been living in a shelter for two weeks. Dolores and Dulce left behind Dulce's older sister and dad in Colombia. They aim to resettle in the United States. Dolores and Dulce completed three sessions (past, present, and future) and completed a total of nine collages across individual and joint formats. Their quotes and/or collages can be found in themes 1 and 2.

Family E consisted of a mother (Esmeralda, 33) and daughter (Elisa, 10) from the Dominican Republic. Esmeralda and her husband had been living in Chile for years and were recently reunited with their children (Elisa and her two older brothers). Yet, the youngest brother is still in the Dominican Republic. The family left Chile in August of 2024 and have been residing in a shelter for three weeks. They aim to resettle in the United States. Esmeralda and Elisa completed three sessions (past, present, and future) and completed a total of nine collages across individual and joint formats. Their quotes and/or collages can be found in themes 1 and 2.

Family F consisted of a mother (Flor, 43) and son (Fernando, 8) from Honduras. They left their home in January of 2024 and have been living in a shelter for six months, with Flor's oldest daughter. Flor is a single mother, and the family aims to resettle in the United States. Fernando has autism, and sessions were adjusted according to their preference to only work

individually. Family F completed all three sessions (past, present, and future) and completed a total of 6 collages. Their quotes and/or collages can be found in themes 1 and 4.

Family G consisted of a mother (Graciela, 39), a daughter (Gina, 12), and son (Gael, 9) from Guatemala. They left their home in October of 2023 and have been living in a shelter for four months, with Gina & Gael's two older brothers and one younger brother. Graciela is a single mother, and the family aims to resettle in the United States. Family G completed two sessions (past and present & future joint session) and completed a total of nine collages across individual and joint formats. Their quotes and/or collages can be found in themes 1, 2, 3, and 4.

Family H consisted of a mother (Hilda, 31) and daughter (Hayley, 10) from Venezuela. They left their home in 2017 and have been living in a shelter for six and a half months, with Hilda's husband and younger son. Hilda was also expecting a baby soon after the session took place. Family H aims to resettle in the United States. They completed one session (all time periods combined) and completed a total of four collages across individual and joint formats. Their quotes and/or collages can be found in theme 4.

Family I consisted of a mother (Irene, 29) and daughter (Itzel, 11) from Venezuela. It is unclear when they left Venezuela, but sometime in 2024. They have resided in the shelter for 50 days. Other members of the family that have made the journey with them include Irene's husband, and three younger children. The three younger children (who were not of age to participate) sat in on the sessions and Itzel was to tend to her siblings. The family aims to return back to Venezuela. Irene and Itzel completed two sessions (past and present & future joint session) and completed six collages across individual and joint formats. Their quotes and/or collages can be found in themes 1, 2 and 4.

Appendix B

Discussion Guide

Session 1: Past

Hello! Thank you so much for joining me today. My name is Madelyn Primeaux, and I'm here as part of my master's research. This project is about learning how children and their mothers experience their migration journey, as well as their expectations for the future. You'll be using art to help tell these stories, focusing on your past, present, and what you imagine for the future.

In these sessions, you'll create collages. A collage is a type of art made from arranging and gluing different materials, like photos, paper, or fabric, onto paper. It allows people to combine different images and textures to create something that can show ideas, memories, or feelings. You can draw, write, paint, or use any of the materials provided. Use what feels right to share your thoughts and feelings. This is your time to be creative and tell your story in your own way.

Everything you share will be kept private, meaning your names and any details that could identify you won't appear in my final project. I'll record our time together to help me remember our conversation, but I'll delete the recording after I write it down, and all names will be changed for privacy.

If you have any questions or if you need to take a break or stop at any time, just let me know.

Before we start with the collage, could each of you share your names, ages, where you're from, when you left your home country, and how long you've been in this shelter?

Thank you! Now, for our first collage, I'd like you to create art that shows an important moment or memory from your journey. For the first 30 minutes, you will work separately, with a divider between you, creating individual parts of the collage—one for the mother and one for the child. The final 30 minutes will be spent working together to create a shared piece that represents your combined memories and stories. I will be here to guide the conversation and ensure you have everything you need, but this artwork is entirely yours to create.

Supportive Questions:

1. "What feelings come to mind when you think about this?"
2. "What images or symbols would you use to show this?"
3. "Who or what is important in this part of your experience?"
4. "What does this make you think about now or in the future?"

Is there anything else you want to add today? Thank you so much for sharing with me. I know your time is valuable, and I really appreciate you taking the time to participate in this project. We'll continue with our next session soon, but if at any point you have questions or concerns, please feel free to reach out to me. If you decide you'd like anything you've shared removed from the project, just let me know within the next two weeks. Do you have any last questions or comments before we finish for today?

Session 2: Present

Welcome back! Today, we'll be focusing on what life is like for you now, here in Mexico. You and your mom will create a collage that shows how the present feels for you. The first 30 minutes will be spent working separately, with a divider between you, to create individual parts of the collage—one for the mother and one for the child. The final 30 minutes will be spent working together to create a shared piece that represents your combined experiences and feelings. I will be here to guide the conversation and ensure you have everything you need, but this artwork is entirely yours to create. Can you create a piece of art that expresses what your life feels like right now?'

Supportive Questions:

1. "What feelings come to mind when you think about this?"
2. "What images or symbols would you use to show this?"
3. "Who or what is important in this part of your experience?"
5. "What does this make you think about now or in the future?"

Thank you both for working on this collage with me today. You did a wonderful job sharing what life is like for you right now. I really appreciate you taking the time to reflect and create together. Before we finish, is there anything else you'd like to add? If you have any questions or thoughts after today, please feel free to reach out. I look forward to seeing you again for our last session, where we'll start thinking about the future!

Session 3: Future

Hello again! For our last session, we'll look toward the future and imagine what life might be like when you reach the United States. You and your mom will create a collage that captures your thoughts and hopes for what's ahead. The first 30 minutes will be spent working separately, with a divider between you, to create individual parts of the collage—one for the mother and one for the child. The final 30 minutes will be spent collaborating to create a shared piece that reflects your combined vision for the future. I will be here to guide the conversation and ensure you have everything you need, but this artwork is entirely yours to create.

Can you create a piece of art that shows what you think life might be like when you reach the United States?

Supportive Questions:

1. “What feelings come to mind when you think about this?”
2. “What images or symbols would you use to show this?”
3. “Who or what is important in this part of your experience?”
4. “What does this make you think about now or in the future?”

Is there anything else you’d like to add before we finish our time together?

Thank you so much for being part of this project and sharing your experiences with me throughout these sessions. Your stories, thoughts, and creativity have been incredibly valuable, and I’m grateful for the trust you’ve shown in participating. If you’d like a summary of my findings once the project is complete, please feel free to reach out. And, if within the next two weeks you decide you’d like anything you’ve shared to be removed, just let me know.

Thank you again for all of your time and insights. Do you have any last questions or comments?

Appendix C

Themes and Codes

24 discussions and 68 collages were included in the analysis. 77 codes were developed from both collages and the discussion. 48 of the 77 codes were used to develop four themes and three subthemes. The following are the 48 codes, their corresponding themes, and their occurrences across collage and discussions.

Theme 1. What Mothers Carry

Subtheme 1. Pain in Partnership

- Code 1: cheating, widowhood, and being left behind, 4 mentions across 4 collages/discussions
- Code 2: lack of support, 4 mentions across 3 collages/discussions

Subtheme 2. Instincts to Protect

- Code 1: child's innocence and unawareness, 4 mentions across 4 collages/discussions
- Code 2: children are past, present and future, 3 mentions across 3 collages/discussions
- Code 3: self-blame, 3 mentions across 3 collages/discussions
- Code 4: sexualizing women (their children), 5 mentions across 5 collages/discussions

Subtheme 3. Reclaiming Selfhood

- Code 1: priorities for mom, 11 mentions across 7 collages/discussions
- Code 2: self-reflection, 33 mentions across 18 collages/discussions

Theme 2. Hardship Beyond Borders

- Code 1: can't return to country of origin, 22 mentions across 14 collages/discussions
- Code 2: current political circumstances, 12 mentions across 7 collages/discussions
- Code 3: danger and threats, 5 mentions across 4 collages/discussions
- Code 4: extortion, 14 mentions across 8 collages/discussions
- Code 5: hunger, 3 mentions across 2 collages/discussions
- Code 6: illiteracy, 2 mentions across 2 collages/discussions
- Code 7: journey to Mexico, 17 mentions across 10 collages/discussions
- Code 8: power dynamics and authority, 9 mentions across 7 collages/discussions
- Code 9: racism, 7 mentions across 4 collages/discussions
- Code 10: suffering and survival, 18 mentions across 9 collages/discussions
- Code 11: making ends meet, 5 mentions across 3 collages/discussions
- Code 12: remittance, 4 mentions across 4 collages/discussions
- Code 13: pets and their connection, 18 mentions across 14 collages/discussions
- Code 14: family left behind, 8 mentions across 7 collages/discussions

Theme 3. Mexico as a Liminal Space

- Code 1: adjusting to Mexico, 8 mentions across 5 collages/discussions
- Code 2: bullying, 4 mentions across 4 collages/discussions

- Code 3: comparing living in country of origin to Mexico, 4 mentions across 3 collages/discussions
- Code 4: feelings about Mexico, 19 mentions across 15 collages/discussions
- Code 5: leaving shelter, 3 mentions across 3 collages/discussions
- Code 6: making ends meet, 5 mentions across 3 collages/discussions
- Code 7: material scarcity, 10 mentions across 9 collages/discussions
- Code 8: Mexico memories, 14 mentions across 10 collages/discussions
- Code 9: migrating within Mexico, 5 mentions across 2 collages/discussions
- Code 10: school in Mexico, 7 mentions across 5 collages/discussions
- Code 11: staying in Mexico, 9 mentions across 6 collages/discussions
- Code 12: thankful for shelter, 11 mentions across 5 collages/discussions
- Code 13: racism, 7 mentions across 4 collages/discussions

Theme 4. A Future Imagined and Fought For

- Code 1: babies (expanding the family), 10 mentions across 7 collages/discussions
- Code 2: careers for kids, 22 mentions across 14 collages/discussions
- Code 3: destination and rationale, 14 mentions across 10 collages/discussions
- Code 4: dream home, 28 mentions across 23 collages/discussions
- Code 5: dreams of travel (without migration), 4 mentions across 4 collages/discussions
- Code 6: driving and learning to drive, 5 mentions across 4 collages/discussions
- Code 7: fear for the future, 2 mentions across 2 collages/discussions
- Code 8: freedom, 3 mentions across 3 collages/discussions
- Code 9: future city, 5 mentions across 5 collages/discussions
- Code 10: imagining the future, 36 mentions across 18 collages/discussions
- Code 11: meaning making, 19 mentions across 17 collages/discussions
- Code 12: mom's career, 7 mentions across 6 collages/discussions
- Code 13: representations of moving forward, 17 mentions across 13 collages/discussions
- Code 14: school in the future, 7 mentions across 6 collages/discussions
- Code 15: stability and normalcy, 19 mentions across collages/discussions

Appendix D

Sample of Materials

Below are photos of some of the materials (cutouts, stickers, scrapbook paper and material).

Participants received a plastic bag for each collage. Each bag that participants received included 9 small paper cutouts, 3 big paper cutouts, 3 glitter stickers, 4 sports stickers, 3 stickers that could be colored, 3 Spanish stickers, 3 random stickers, sequin stickers, 1 piece of material, and 1 scrapbook paper. These materials were randomly sorted and placed in the bags to ensure participants had access to the same sorts of things in each session. Another constant in each session were the curated images in Appendix D.

If more materials were needed, they were available upon request. Furthermore, participants were still allowed to use the printer if they felt they were missing anything. Participants were given 12 sheets of colored paper each session to choose what color they would like best for the collage.

9 small cutouts & Scrapbook Paper and Material

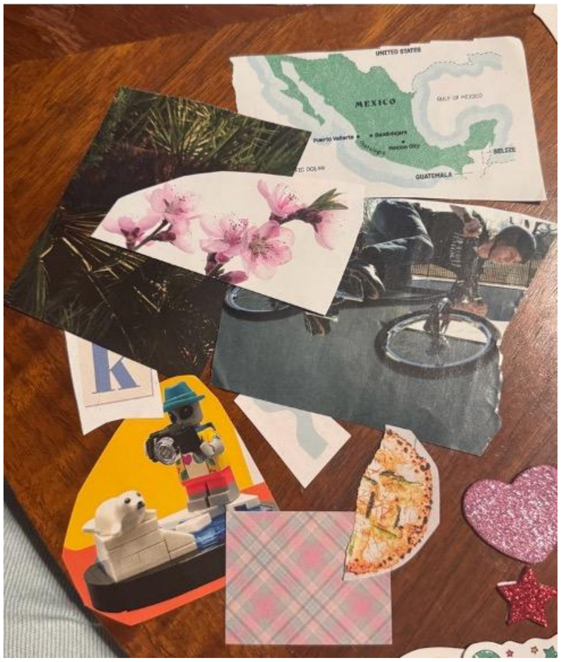

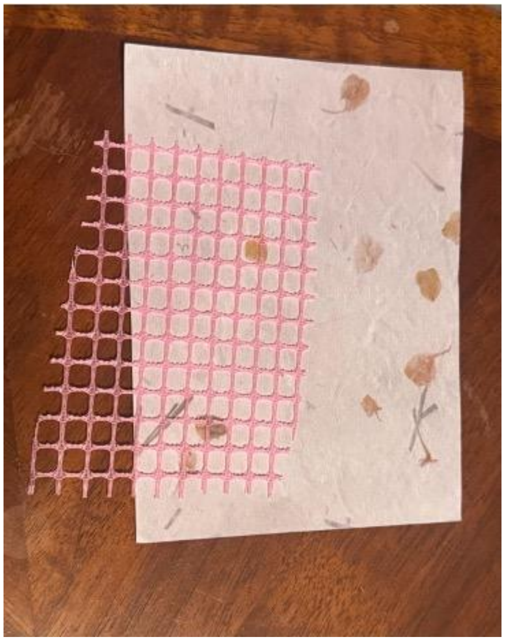

3 Large Cutouts & Stickers

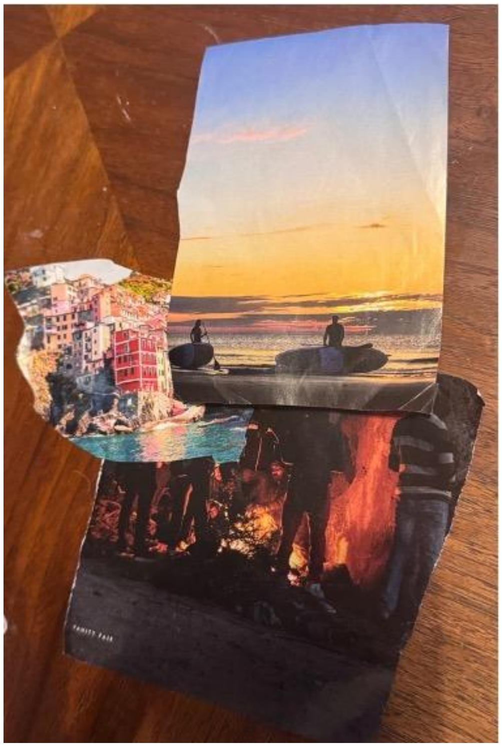

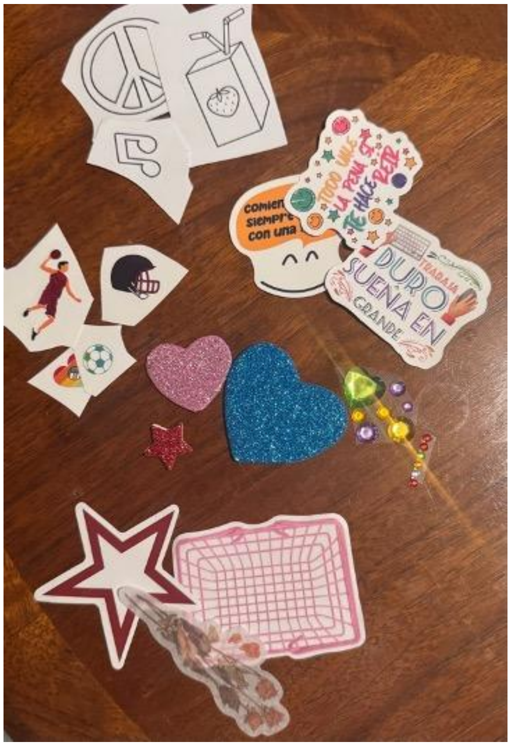

Appendix E
Curated Images

Explanation

In each collage session, participants received multiple copies of the following images. In selecting the imagery for the collages, I drew on existing research and projects involving children, art, and migrant communities. These projects utilized art and world-building exercises as methods of understanding migrant narratives (UNICEF Eastern Caribbean Area, 2019; Marchevska & Defrin, 2023). Prior research and projects have shown that these types of visual and participatory methods allow individuals to convey complex themes related to displacement, belonging, and identity (Brown et al., 2020; Garriba, 2024; UNICEF, 2023).

Several studies found common themes in children's artistic expressions. For example, symbols such as flags and boats prominently appeared in children's drawings related to their journeys through the Darien Gap (UNICEF, 2022). Similarly, a sand play study with refugee children highlighted the frequent use of figures representing people and military personnel to express narratives (Kronick et al., 2018). This finding aligns with an article from *The New York Times* on art in detention camps, which also noted the significance of religious symbols in children's art (Brown, 2019). The sand play study further documented depictions of buildings, such as churches, schools, houses, and modes of transportation, planes, trains, busses, findings that were echoed in art-based narrative research conducted in South Africa and in drawings from the Darien Gap (Kronick et al., 2018; Brown, 2019; Clacherty, 2021).

Other studies reinforced these patterns. Houses were consistently observed in children's drawings analyzed by Farokhi and colleagues (Farokhi & Hashemi, 2011). Toys were another recurring theme, seen in both Flora Thompson's documentation of children carrying cherished items to safety in the UK and in my own observations at the shelter (Thompson, 2022). Fences were significant in the sand play study and appeared similarly in the South Africa study, which also noted the importance of suitcases, backpacks, and symbols related to financial security, such as money, as representations of hope and survival (Kronick et al., 2018; Clacherty, 2021). These repeated findings across different studies and projects highlight common elements in children's representations of their migration experiences, underscoring shared themes of resilience, displacement, and identity.

Curated Images (Flag, School, House and Church)

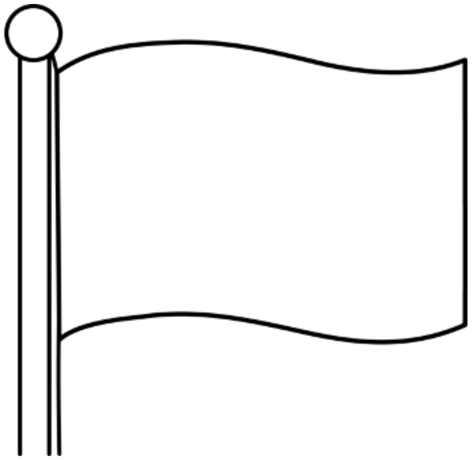

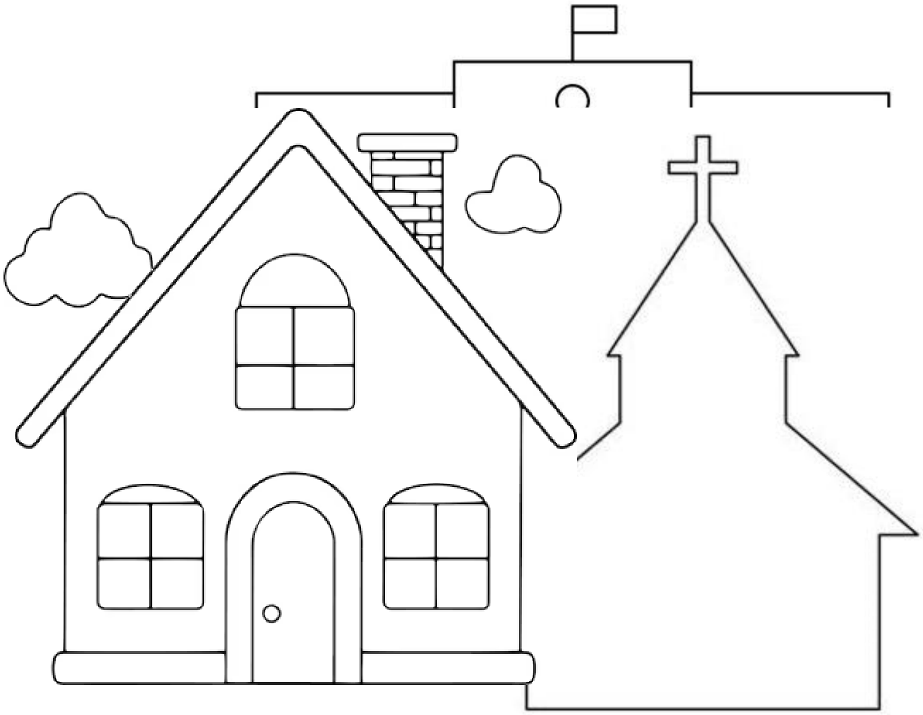

Curated Images

Transportation (Bus, Boat, Train, Airplane)

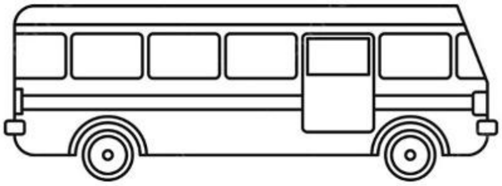

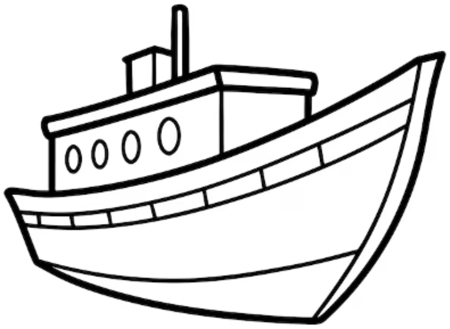

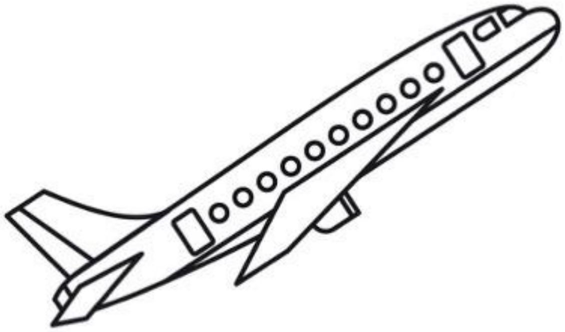

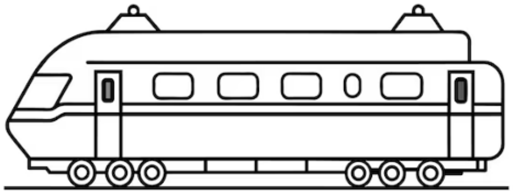

Curated Images
(Different people figures, law enforcement, money)

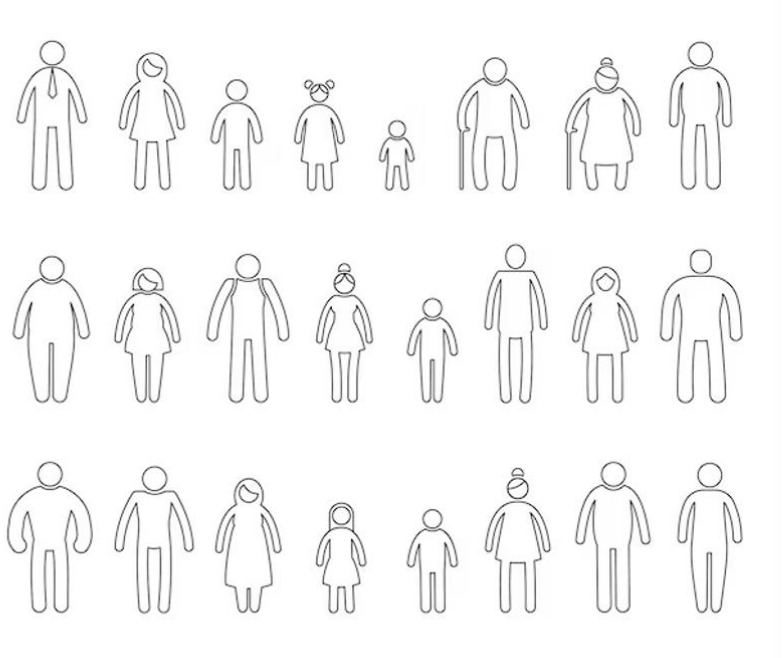

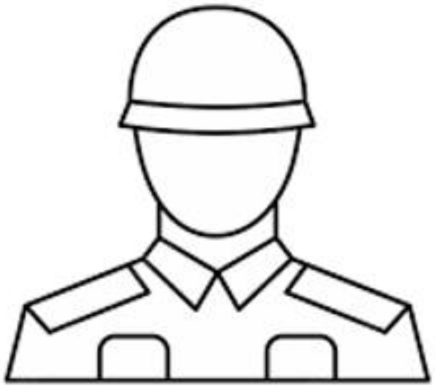

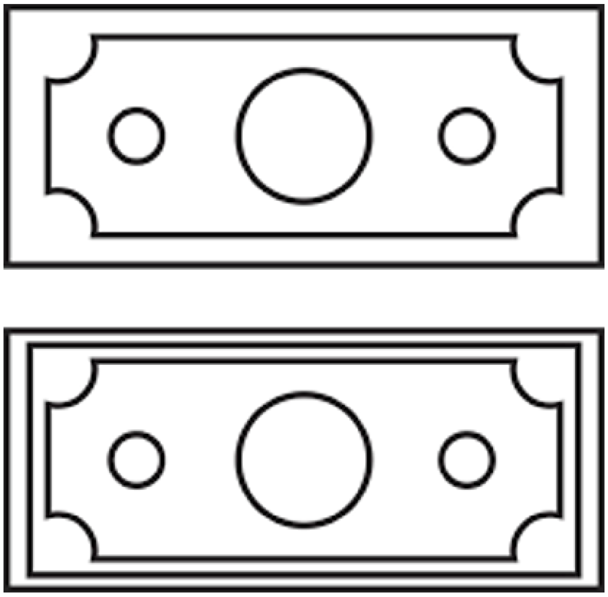

Curated Images

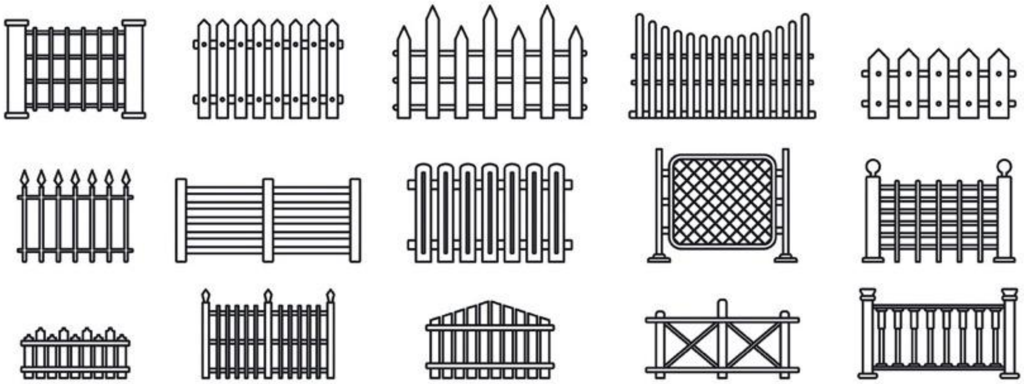

Fences and Religious Symbols

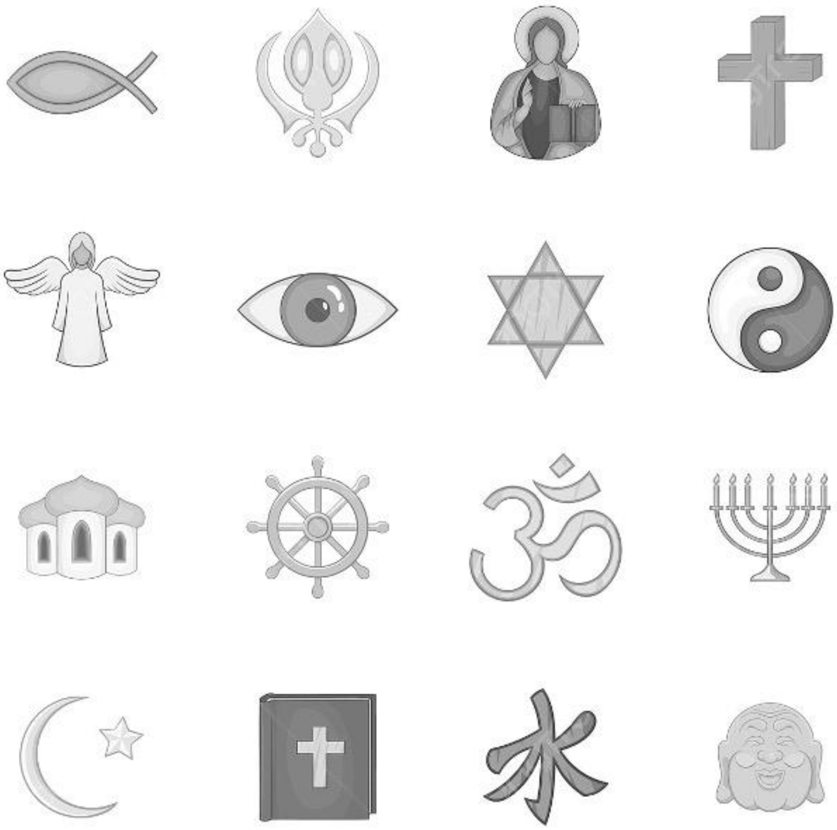

Curated Images

(Toys and Luggage: Backpack/Suitcase)

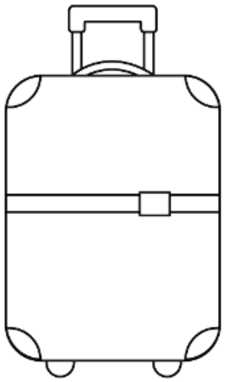

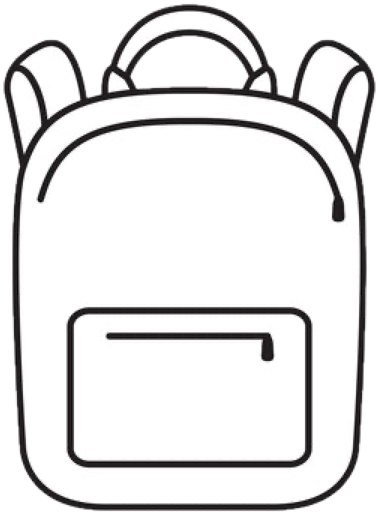

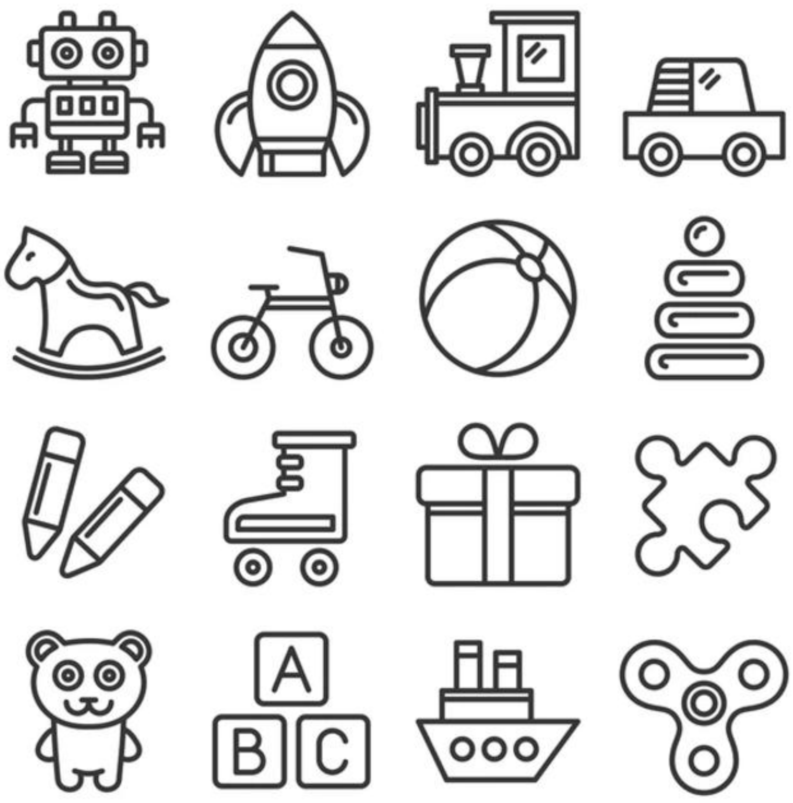
A
B
C

Appendix F
Continuation of Colonialism

This legacy dates back to the 1823 Monroe Doctrine that opposed further European colonization but positioned the United States as the dominant power of the Americas (Coronado, 2019). In Venezuela, the U.S. supported the 2002 coup against Chávez and imposed sanctions that deepened the country's economic collapse and humanitarian crisis (Páez, 2017; Ribando Seelke et al., 2022). In Honduras, it legitimized the 2009 coup against President Zelaya and has continued to fund militarized security forces linked to violence and repression, contributing to the very instability that drives migration (The Center for Justice and Accountability). Understanding the stories shared by migrant families requires us to recognize these histories and the ongoing systems that shape their realities today.
